# Promotion of the influenza vaccination to hospital staff during pre-employment health check: a prospective, randomised, controlled trial

**DOI:** 10.1186/s12995-020-00285-w

**Published:** 2020-11-18

**Authors:** Michael Currat, Catherine Lazor-Blanchet, Giorgio Zanetti

**Affiliations:** 1 Service of Hospital Preventive Medicine, Lausanne University Hospital, Occupational Medicine Unit BH-08, Rue du Bugnon 46, CH-1011 Lausanne, Switzerland; 2 Faculty of Biology and Medicine, University of Lausanne, CH-1015 Lausanne, Switzerland

**Keywords:** Influenza, Vaccine, Healthcare workers, Vaccination coverage, Occupational health service

## Abstract

**Background:**

Vaccination is the most effective prevention of seasonal influenza. Despite its recommendation and active promotion, vaccination coverage remains low among healthcare staff. The goal of the study was to test if a pre-employment health check is a good opportunity to promote future vaccination against influenza among healthcare workers newly hired by a university hospital.

**Methods:**

All new hospital employees active at the bedside who underwent a pre-employment health check between the end of 2016’s influenza epidemic and the start of the next influenza vaccination campaign were randomly allocated to a control group or an intervention group. The intervention consisted of a semi-structured dialog and the release of an information leaflet about influenza and influenza vaccination during the check-up, and the shipment of a postcard reminder 2 weeks before the next vaccination campaign. Vaccination rates during the campaign were compared among the two groups.

**Results:**

Three hundred fifty-seven employees were included. Vaccination rates were similar in both groups: 79/172 (46%) in the control and 92/185 (50%) in the intervention group. A significantly higher rate of vaccination was noted among physicians (70/117, 60%) than among other employees (101/240, 42%, *p* = 0.001). In a pre-defined exploratory analysis among physicians, the vaccination rate was higher in the intervention group (36/51, 71%) than in the control group (34/65, 52%, *p* = 0.046).

**Conclusions:**

Promotion of the influenza vaccine during pre-employment health check did not improve the vaccination rate of newly hired hospital healthcare workers overall during the next influenza vaccination campaign. Results suggest a favourable impact on the vaccination rate of physicians. Thus, there may be an interest in using communication strategies tailored to the different categories of healthcare workers to promote the influenza vaccine during pre-employment health check.

**Trial registration:**

ClinicalTrials, NCT02758145. Registered 26 April 2016.

**Supplementary Information:**

The online version contains supplementary material available at 10.1186/s12995-020-00285-w.

## Background

Influenza is an infectious disease responsible for a significant number of hospitalisations and deaths every year during its seasonal epidemic period [1–3]. Vaccination against seasonal influenza is the most effective means of preventing infection [4]. It is specifically recommended for staff in healthcare institutions who are in regular contact with patients. This recommendation is supported by global and national health authorities, such as the World Health Organization [5], the USA’s Center for Disease Control [6] and Switzerland’s Federal Office of Public Health [7]. Several studies carried out in chronic care institutions for the elderly have shown that reduced mortality rates among residents were directly related to healthcare staff’s vaccination against influenza [8–10]. According to one model, the benefits of vaccination in acute care institutions would be the same as those observed in chronic care institutions for the elderly, if not superior [11]. Healthcare institution employees who are in regular contact with patients are intensively exposed to influenza, with infection rates of over 20% during epidemic periods [12], and significant consequences in terms of disease transmission to patients and absenteeism [13].

Influenza vaccination rates in healthcare workers generally remain low, varying from 5 to 54% (mean 24%), in European countries which, like Switzerland, do not make it mandatory [14, 15]. Several elements contributing to this lack of coverage have been identified. Staff vaccination rates are lower when they perceive influenza to be a benign disease with regards to themselves or their families, when they do not believe themselves to be at risk of catching the disease, when they are afraid about the vaccination’s potential adverse effects or have doubts about its efficacy, or when the vaccination itself is difficult to access. Fears about injections and the need to get a repeat vaccination annually also have unfavourable effects on staff vaccination rates [15–17].

A variety of complementary actions are recommended for promoting better rates of vaccination coverage among hospital personnel, among which are easy access to vaccination, free vaccination, management commitment, in-house communication and personnel awareness-raising programmes (posters, intranet messages, in-house magazines, seminars, etc.) [18–20]. A targeted awareness-raising approach aimed towards newly hired hospital personnel could thus be particularly valuable. As these new staff must undergo a pre-employment health check, which generally involves verification of immunological status and vaccination booster injections if necessary, this would seem to be a good opportunity to give out information about influenza and its vaccine.

The present study’s aim was therefore to measure the impact of the standardised information given to new hospital staff about influenza and its vaccine on the rate of vaccination by those same staff during the institution’s next annual seasonal vaccination campaign.

## Methods

This monocentric, prospective, randomised, controlled trial took place in a tertiary, 1522-bed university teaching hospital with 11,039 employees, in the French-speaking region of Switzerland. All employees aged ≥18 years old, who underwent a pre-employment health check between the end of 2016’s influenza epidemic and 31 October 2016 (the eve of the start of the next influenza vaccination campaign) and who directly delivered care or services to patients were eligible for participation in the study. It should be noted that employees who underwent a pre-employment health check may have been starting their first job in the hospital, have been re-hired or may have been current employees who were taking on a new professional role or departmental posting. No exclusion criteria were applied.

The employees included in the study were randomly allocated 1:1 to one of two parallel groups based on a computer-generated seven-digit identification number that is attributed to each one on hiring, by order of commencement of their duties, regardless of all other parameters. The intervention and control groups were thus made up of the odd-numbered and even-numbered employees, respectively. The control group underwent the usual pre-employment health check only. The intervention group also underwent an additional three-phase intervention. During the health check, as phase one, a nurse experienced in immunisation gave a short, standardised briefing about the issues at stake surrounding influenza, current recommendations about influenza vaccination and the hospital’s annual vaccination campaign, as well as a semi-structured interview about any previous influenza vaccinations the new employee might have had. The nurse then answered any questions which the employee might have, referring to a physician if necessary. Immediately after the interview, as phase two, the nurse gave new employees an information leaflet including key messages, answers to frequently asked questions, links to internet sites of reference and a contact address in case of any further questions (see Additional file 1). The nurse also informed them that they would receive a reminder by mail about this information at the start of the next annual seasonal influenza vaccination campaign. Phase three, at the start of the vaccination campaign, involved sending those employees an informative postcard reminder (see Additional file 2) about the material given to them during their pre-employment health check and inviting them to get vaccinated for free at one of the various dedicated locations around the hospital campus. The contents of all this documentation were designed in collaboration with communication specialists; they were evidence-based, as they took into account data from the literature on the determinants of influenza vaccination uptake among healthcare workers [16, 21, 22].

The hospital-wide vaccination campaign against seasonal influenza (starting 1 November 2016) continued until the end of the seasonal influenza epidemic (on 25 February 2017 in Switzerland). Not only did the Occupational Health Unit offer free vaccination against seasonal influenza to all hospital staff but it was also set up at a series of provisional vaccination points in highly frequented areas around the hospital campus and delegated vaccinators in each major department. The campaign was backed by a communication and awareness-raising programme using posters, posts on the hospital intranet and a series of educational video messages which could be uploaded onto smartphones by scanning the QR code on stickers located throughout the institution. It should be noted that during the seasonal influenza epidemic, unvaccinated personnel working in care units are required to wear a surgical mask at all times.

Data on age, sex, nationality, marital status, profession, previous employment in the hospital, ongoing training, self-reported asthma or allergies, and administration or not of the influenza vaccine during the next influenza vaccination campaign, were extracted from employees’ computerised medical records. Nationalities were categorised as Swiss or others, and professions were categorised as physicians, patient-care employees (including nurses, nursing and care assistants, physiotherapists and occupational therapists) and others (technical, logistical, administrative and psychosocial personnel).

Employees initially included in the study who subsequently left the hospital before the start of the influenza vaccination campaign were lost to follow-up and were thus excluded from the final analysis. Sampling was defined pragmatically so as to include all the staff eligible between the end of one year’s seasonal influenza epidemic and the start of the next influenza vaccination campaign. On the basis of previous years’ data, the sample was estimated at 400 new employees. By applying the average vaccination coverage rate of 45% to the control group (the coverage rate observed across all hospital staff in the year before the study), a sample of 200 employees in each group would give the study a power of 0.85 to detect a significant 60% difference in vaccination coverage in the intervention group. The primary assessment criteria were the rates of influenza vaccination in the intervention and control groups. Exploratory comparisons were also made between subgroups of employees. Statistical analyses were made using STATA 15.0 software (StataCorp, College Station, Texas 77,845). Continuous variables were compared using the Wilcoxon rank sum test, and proportions were compared using the chi-squared test or Fisher’s exact test, as appropriate. All tests were bi-directional. A multivariate logistic regression was also performed to take into account any potential residual disequilibria between groups. Covariables were selected using a stepwise process which maintained the group in the model. Excluded covariables were then tested as potential confounding factors in the final model. A value of *p* < 0.05 was considered to be statistically significant.

This study was approved by the Human Research Ethics Committee of the Canton Vaud, BASEC number 2016–00296, and was registered in the American database of clinical trials, www.clinicaltrials.gov, as NCT02758145.

## Results

**Fig. 1:**
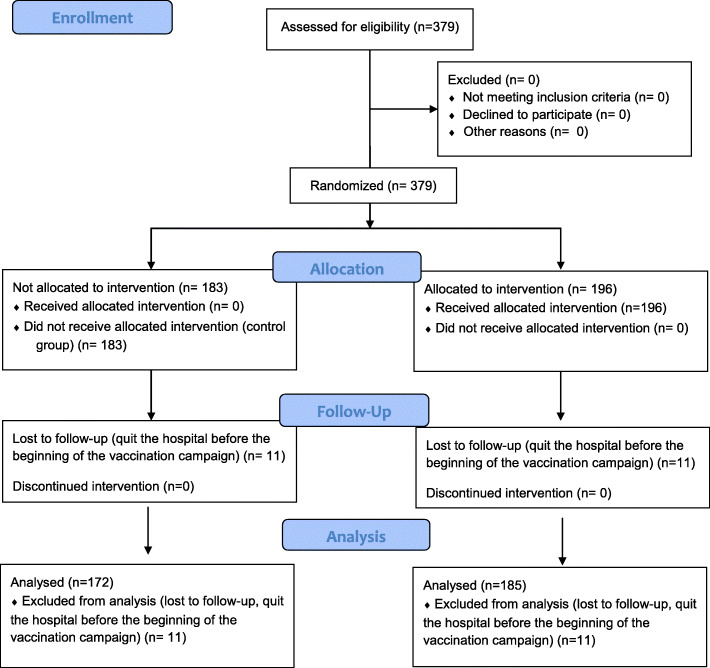
Study flow diagram

The study protocol included a total of 379 employees: 183 in the control group and 196 in the intervention group (Fig. 1). Twenty-two employees (< 1%), 11 from each group, were lost to follow-up and were thus excluded from our analysis. The two groups’ demographic characteristics are shown in Table 1. Despite randomisation, mean age was significantly lower and the proportion of physicians was significantly higher in the control group than in the intervention group.

**Table 1:** Study population characteristics

Characteristics	Control group (*n* = 172)	Intervention group (*n* = 185)
Mean age, years (standard deviation)	33.0 (8.1)*	31.3 (7.3)*
Female sex, n (%)	118 (69)	139 (75)
Married, n (%)	39 (23)	45 (24)
Swiss nationality, n (%)	89 (52)	95 (51)
Allergies, n (%)	49 (29)	52 (28)
Asthma, n (%)	11 (6.4)	9 (4.9)
New employee, n (%)	81 (47)	106 (57)
Staff undergoing training, n (%)	6 (3.5)	15 (8.1)
Physician, n (%)	65 (38)*	51 (28)*
Patient-care employee, n (%)	72 (42)	86 (47)
Other personnel, n (%)	35 (20)	48 (26)

* *P* value < 0.05

Table 2 compares vaccination rates as a function of the variables observed. In the control group, 79/172 (46%) employees were vaccinated during the vaccination campaign which followed their inclusion in the study; the proportion was 92/185 (50%) in the intervention group, a difference that was not significant. A significantly higher rate of vaccination was noted among physicians than among other professions, and that rate was significantly lower among staff undergoing training and professions other than physicians or patient-care employees. Furthermore, we noted that vaccination rates were close to being significantly higher among staff who stated that they were asthmatic or who were considered new employees. The mean age was identical (32.1 years) among the employees who did and did not get vaccinated.

**Table 2:** Vaccination rate by enrolled employees’ characteristics

Characteristic	n (%) of vaccinated employees among those with the characteristic	n (%) of vaccinated employees without the characteristic
Intervention group	92 (50)	79 (46)
Female sex	123 (48)	48 (48)
Married	46 (55)	125 (46)
Swiss nationality	88 (48)	83 (48)
Allergies	54 (54)	117 (46)
Asthma	14 (70)	157 (47)
New hospital employee	98 (52)	73 (42)
Staff undergoing training	5 (24)*	157 (48)*
Physician	70 (60)**	101 (42)**
Patient-care employee	75 (48)	103 (51)
Other personnel	26 (31)**	145 (53)**

* *P* value < 0.05; ** *P* value < 0.01

According to the multivariate analysis (Table 3), being a physician, self-reported previous asthma and being a new employee appeared to be independent predictors of getting an influenza vaccination. Being a healthcare professional other than a physician or a patient-care employee was a predictor of non-vaccination. The intervention’s impact, however, was not significant.

**Table 3:** Multivariate analysis of the predictors of influenza vaccination

Variable	Odds Ratio	95% Conf. Interval
Intervention group	1.26	0.81–1.96
Self-reported asthma	2.92	1.03–8.26
New hospital employee	1.88	1.19–2.95
Physician	1.87	1.13–3.10
Other personnel	0.44	0.25–0.79

Several subgroups underwent an exploratory analysis. The influenza vaccination rate among physicians in the intervention group was significantly higher (36/51, 71%) than that for physicians in the control group (34/65, 52%, *p* < 0.05), corresponding to a relative risk of 1.35 (95%CI 1.01–1.81). For this subgroup, the intervention’s positive impact on the vaccination rate remained close to statistical significance in the multivariate analysis (OR 2.10, 95%CI 0.94–4.69). The analyses in the other subgroups (see Additional file 3) didn’t yield interesting results.

## Discussion

In this randomised study conducted in a tertiary university teaching hospital, an awareness-raising intervention about influenza vaccination during the pre-employment health checks was not associated with any significant modification in vaccination coverage during the next campaign of vaccination against seasonal influenza. Results did, however, suggest the positive impact of awareness-raising on physicians, with a vaccination coverage rate moving from 52 to 71%.

The intervention consisted of a standardised oral briefing given by a nurse during personnel’s pre-employment health check. This was accompanied by written documentation and an invitation for free vaccination in the form of a postcard sent to the staff member’s home at the start of the next seasonal influenza vaccination campaign. To the best of our knowledge, no such intervention has been previously described. Furthermore, the present study is one of few to use a randomised, controlled design to evaluate the impact of measures encouraging influenza vaccination in healthcare personnel. Indeed, the majority of published studies on this topic used quasi-experimental before-and-after-type designs. However, that type of design is more subject to confounding, and it can be influenced by variations in the severity of seasonal influenza epidemics and the population’s perceptions of the disease.

Several studies have shown that programmes comprising of numerous complementary interventions (free vaccinations, flexible workplace vaccine delivery, educational materials, etc.), strong commitment by management and leaders, and personnel specifically dedicated to the programme can indeed improve influenza vaccination coverage among hospital staff [23–25]. Despite these findings, vaccination coverage rates generally remain low, with a mean of 24% in European countries [14]. Research on innovative complementary approaches to improve coverage via studies such as the present one remains important.

The present study’s negative overall results may seem surprising considering that each new employee underwent an individual consultation with an occupational health nurse—an occasion which seems ideally suited to promote vaccination. Our findings showed that this measure did not deliver a significant enough stimulation. We could also hypothesise that, for a large proportion of the population studied, the first phase of the intervention came long before staff needed to decide about whether or not to be vaccinated. Additionally, the intervention’s positive impact on the subgroup of physicians suggests that measures may need to be better adapted to different subgroups of hospital employees. Finally, except for mandatory vaccination [23, 24, 26], literature suggests that an isolated, individual intervention measure is unlikely to increase vaccination coverage rates in any significant way [23–25, 27–29].

The physicians included in the present study reached a significantly higher vaccination rate (60%) than other employees (42%). Several studies have had similar findings [28, 30]. Furthermore, an analysis limited to the subgroup of physicians suggested that the intervention significantly increased that population’s rate of vaccination by 35%. Although the intervention’s impact on the subgroup of physicians was close to statistical significance in the multivariate analysis, this result should be considered carefully as it was not the study’s primary objective. It does, nevertheless, suggest that the intervention could have a different impact on different healthcare professions. Indeed, this hypothesis is corroborated by a 2017 study carried out in a South Korean tertiary hospital [31] that also showed that individual, person-to-person awareness-raising was associated with an increased vaccination coverage rate among physicians only. A range of studies have shown the need to provide specific messages adapted to different professional groups as each category has different representations of, and attitudes towards, seasonal influenza vaccination [23, 24, 32]. With this in mind, future research should examine how to differentiate awareness-raising according to the specific target audiences taking part in pre-employment health checks.

This study had some limitations. Firstly, its statistical power was limited by the necessity to include a pragmatic sample of the employees invited to their pre-employment health check between the end of one seasonal influenza epidemic and the start of the vaccination campaign for the next one. It would have been ethically inexcusable to have a control group that would not receive any information during the seasonal influenza epidemic or during the vaccination campaign. We also had a smaller potential sample because the seasonal influenza epidemic which preceded our study finished late (second half of April 2016). Furthermore, the study took place within the context of a significant overall increase in the total hospital seasonal influenza vaccination coverage. The rate went up to 51% during the vaccination campaign included in our study, compared to 43% 2 years before. This overall trend may well have reduced the probability of identifying our intervention’s impact on new personnel.

Secondly, randomisation did not allow a perfect balance between the two experimental groups in terms of age, profession and professional experience within our institution. Nevertheless, our multivariate analysis did not suggest that this imbalance could have masked any significant interventional impact.

Thirdly, it was impossible to guarantee total independence between the two experimental groups. Personnel in the intervention group could easily have spoken about their health check experience to personnel in the control group, thus constituting a contamination of the control group that would attenuate the intervention’s measurable impact. We believe the potential for such a bias is small, however, because the 357 personnel included in the study were spread across a university hospital campus with over 10,000 employees.

Fourthly, there was no pilot phase to test the readability and intelligibility of the information material used. Although these materials were developed with communication specialists, their use in a pilot phase would have ensured that they were understandable to all study participants.

Finally, the primary outcome indicator (influenza vaccination during the seasonal influenza vaccination campaign which followed enrolment in the study) could have been affected by an observation bias if employees were vaccinated outside of the institution and were therefore falsely considered as unvaccinated. It is doubtful, however, that such a potential bias played a significant role in our results as influenza vaccinations are administered for free across our institution but have to be paid for elsewhere.

## Conclusions

An intervention during the pre-employment health check of newly employed hospital personnel had no significant effect on the ensuing influenza vaccination coverage rate. However, the results suggest a favourable impact on the coverage rate in the subgroup of physicians. Consequently, similar interventions promoting information and briefings specifically tailored to different categories of healthcare professionals deserve further research. The success of programmes aimed at improving the influenza vaccination coverage rates among hospital personnel rests primarily on combining complementary and synergistic actions that should be implemented over the long term. 

## Supplementary Information

**Additional file 1.** Information leaflet.

**Additional file 2.** Informative postcard reminder.

**Additional file 3.** Exploratory comparisons in other subgroups.

## Data Availability

The datasets used and/or analysed during the current study are available from the corresponding author on reasonable request.
