# Supplementary material for: Promotion of the influenza vaccination to hospital staff during pre-employment health check: a prospective, randomised, controlled trial
Source: J Occup Med Toxicol. 2020 Nov 18;15:34. doi: 10.1186/s12995-020-00285-w (PMC7672907; doi:10.1186/s12995-020-00285-w)
Supplement: Supplementary file 1 — Additional file 1. Information leaflet. [file 12995_2020_285_MOESM1_ESM.pdf]

Le saviez-vous?

Durant une épidémie de grippe saisonnière,
chaque année, en Suisse, plus d'

1 million de personnes

vont attraper la grippe dont

300'000 enfants.

25% du personnel soignant

peut être contaminé par la grippe.
Cela va engendrer plus de

150'000 consultations,

jusqu'à

5'000 hospitalisations

et

1'500 décès

Source: OFSP, septembre 2011.

Pour plus d'informations, consultez :

Site de l'OFSP pour la population en général
www.sevaccinercontrelagrippe.ch

Site de l'OFSP pour les professionnel·le·s de la santé
www.grippe.admin.ch

Informations sur les vaccins en général et aussi sur le vaccin contre la grippe
www.infovac.ch

Centre national de référence de l'influenza, Genève
www.influenza.ch

Nous sommes aussi à votre disposition en cas de questions :

Unité de médecine du personnel et d'entreprise du CHUV
Bâtiment hospitalier principal
Niveau 8
021 314 02 43
medecine.personnel@chuv.ch

SAM | CHUV 2016 | 18877

canton de vaud

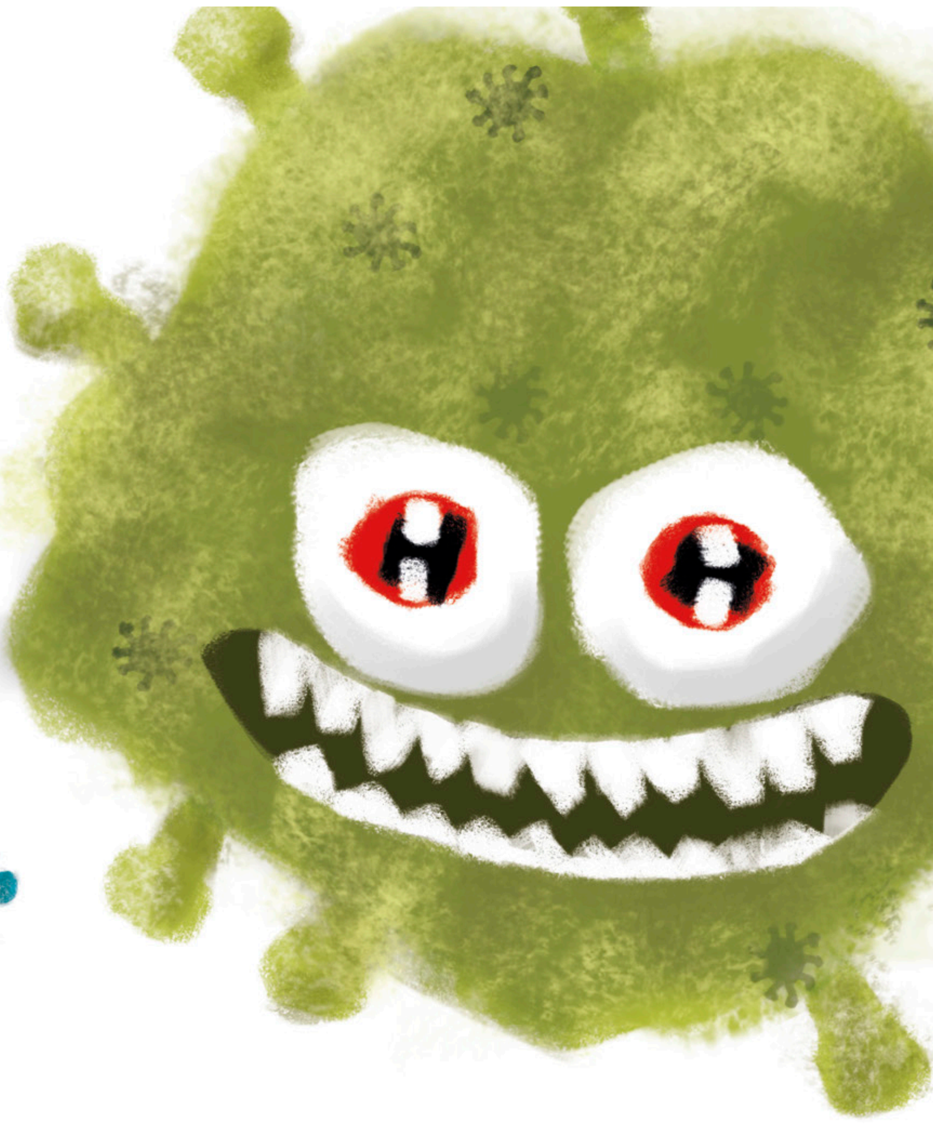

Médecine du personnel

Ne vous trompez pas d'ennemi ! Méfiez-vous de la grippe, pas du vaccin.

CHUV

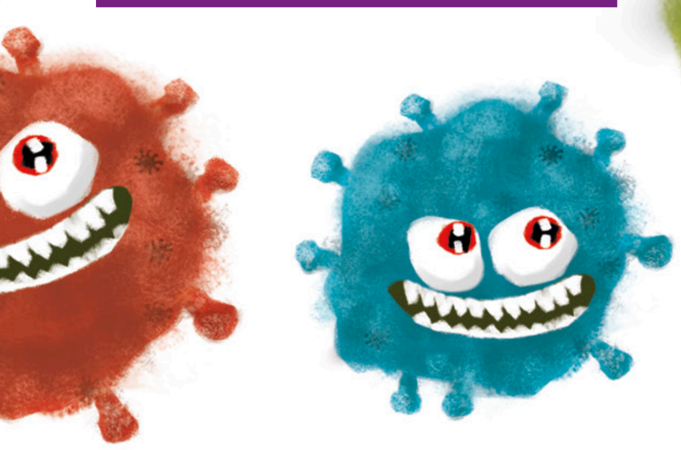

Se faire vacciner contre la grippe… mais pourquoi ?

Je reste en forme.

En travaillant dans un hôpital, je suis plus à risque d'attraper la grippe que d'autres personnes. Le CHUV et la PMU m'offrent le vaccin dès le début du mois de novembre et durant toute l'épidémie. Grâce à lui, je passerai mes vacances de février sur les skis et pas au lit !

J'évite de transmettre le virus à d'autres personnes à la santé plus fragile.

La grippe, très contagieuse, n'est pas une maladie anodine. Ses complications peuvent être sévères et entraîner le décès des personnes à risque. En me vaccinant, je diminue le risque de transmettre la grippe aux patients que je côtoie et je préviens la survenue d'épidémies de grippe à l'hôpital. Je protège aussi mon entourage à la santé fragile: par exemple ma femme enceinte, mon nouveau-né, mon conjoint qui a une maladie chronique, mes parents de plus de 65 ans, etc.

Le vaccin est une méthode sûre et efficace !

En aucun cas le vaccin ne peut transmettre le virus de la grippe car il ne contient pas de virus ! S'il peut occasionner parfois quelques symptômes (douleurs et rougeur au site injection, plus rarement fièvre et sensation de mal-être), ceux-ci sont passagers. Les complications graves suite à une vaccination sont bien plus rares que celles dues à la grippe.

Je n'ai pas besoin de porter le masque à l'hôpital.

Deux semaines après le vaccin, je suis immunisé·e. Durant l'épidémie de grippe, je n'ai donc pas besoin de porter un masque dans les services de soins, sauf si je présente des symptômes respiratoires.

Stop aux idées reçues !

La grippe n'est pas une maladie grave.

FAUX

La grippe, très contagieuse, provoque le plus souvent des symptômes pénibles, tels que forte fièvre, courbatures, maux de tête, toux importante pendant plusieurs jours avec une période de convalescence qui peut durer plusieurs semaines. De plus, elle peut être la cause de complications respiratoires (pneumonies), cardiaques (myocardites), neurologiques (encéphalites, méningites) ou aggraver une maladie préexistante (diabète, insuffisance cardiaque ou respiratoire, etc.). Elle provoque chaque hiver de nombreux décès chez les personnes à la santé fragile.

Je ne suis jamais malade. Le vaccin ce n'est pas pour moi.

FAUX

Tout le monde peut attraper la grippe. Même chez les personnes en bonne santé, elle peut entraîner des complications. En étant malade, on augmente par ailleurs le risque de transmettre le virus aux personnes de notre entourage privé ou professionnel qui ont une santé plus fragile (personnes âgées, femmes enceintes, enfants de moins de 6 mois, enfants prématurés, personnes avec maladie chronique, etc.).

Le vaccin, c'est seulement pour les personnes malades, les personnes âgées.

FAUX

Les personnes malades ou âgées sont moins bien protégées par le vaccin (efficacité entre 25% et 50%). C'est pour cela que le vaccin est recommandé pour toutes les personnes qui côtoient dans leur vie privée ou professionnelle des personnes à risque de complications.

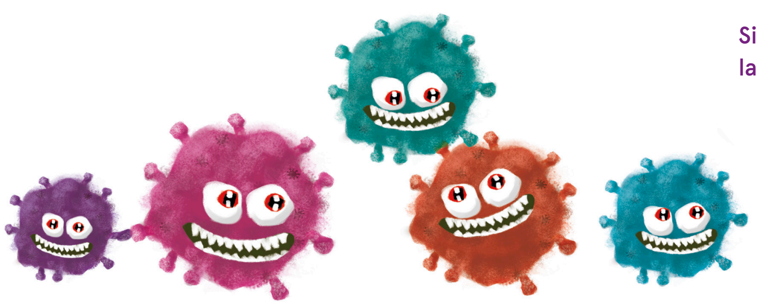

L'année passée, je me suis vacciné et j'ai attrapé la grippe. Le vaccin n'est pas efficace et ne sert donc à rien.

Principalement FAUX

Il faut environ 2 semaines pour que le vaccin soit efficace, il est donc possible d'attraper la grippe durant cette période. Par ailleurs, le vaccin ne protège que contre la grippe et pas contre les autres virus qui circulent aussi durant l'hiver et qui sont responsable de symptômes ressemblant à ceux de la grippe. Il faut savoir aussi que les virus de la grippe se modifient fréquemment et les souches contenues dans le vaccin sont choisies 6 mois avant l'épidémie. Il peut donc aussi arriver qu'il y ait une légère différence entre les souches vaccinales et un virus circulant. Dans ce cas, le vaccin peut être moins efficace.
Même s'il n'est pas parfait, le vaccin reste le meilleur moyen de prévenir la maladie, en protégeant entre 70% et 90% les personnes en bonne santé. Chez les personnes fragilisées par la maladie ou l'âge, il est moins efficace mais permet de diminuer la durée et la gravité de la maladie.

Le vaccin diminue les défenses immunitaires.

FAUX

Le vaccin va au contraire stimuler le système immunitaire pour qu'il puisse par la suite être plus efficace contre le virus. Il ne diminue pas les défenses contre les autres infections.

Le vaccin contient des substances dangereuses.

FAUX

Le vaccin ne contient aucune substance dangereuse. Il est seulement contre-indiqué en cas de réaction allergique grave (choc anaphylactique) à l'un de ses composants. Le vaccin ne doit pas être administré lors d'une allergie grave connue aux protéines de l'œuf. C'est pour cela que le médecin ou l'infirmière se renseigne sur les antécédents allergiques avant toute vaccination. Le vaccin utilisé au CHUV et à la PMU ne contient pas d'adjuvant.

Si je mets un masque chaque que fois que je travaille, je protège mes patients aussi bien que si je me vaccine.

FAUX

Si le port du masque peut réduire la transmission de la grippe, la vaccination reste le moyen de prévention le plus efficace.
