# Supplementary material for: Promotion of the influenza vaccination to hospital staff during pre-employment health check: a prospective, randomised, controlled trial
Source: J Occup Med Toxicol. 2020 Nov 18;15:34. doi: 10.1186/s12995-020-00285-w (PMC7672907; doi:10.1186/s12995-020-00285-w)
Supplement: Supplementary file 2 — Additional file 2. Informative postcard reminder. [file 12995_2020_285_MOESM2_ESM.pdf]

Médecine du personnel

Ne vous trompez pas d'ennemi ! Méfiez-vous de la grippe, pas du vaccin.

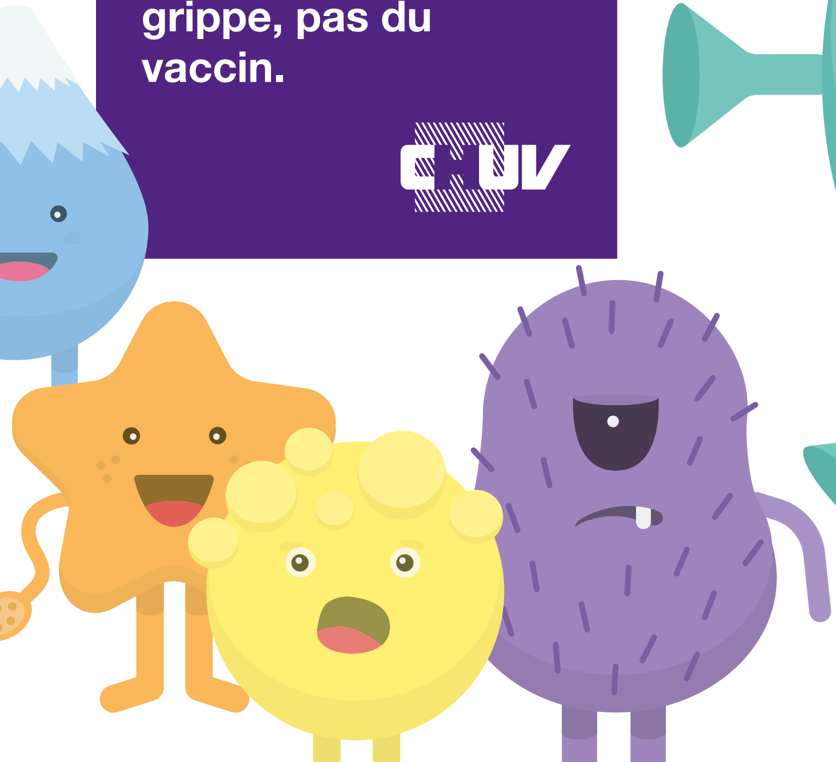

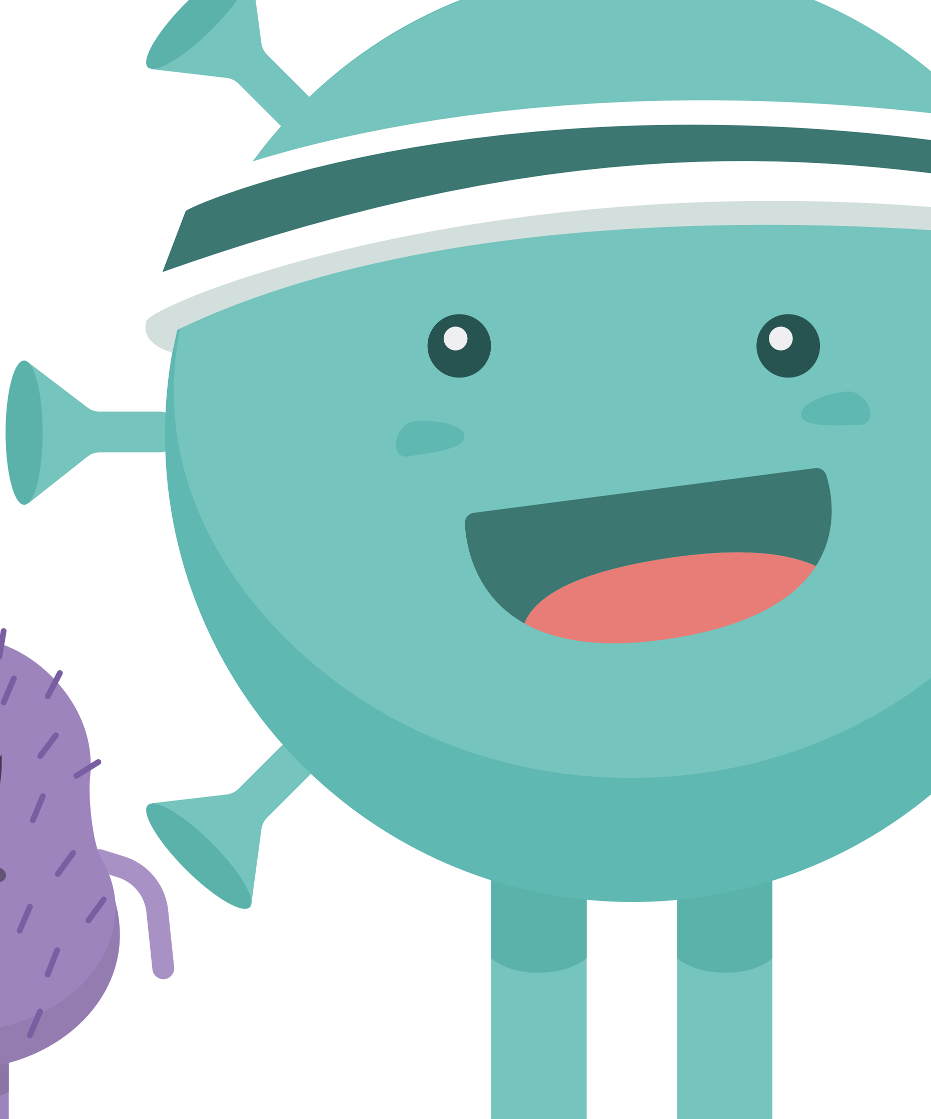

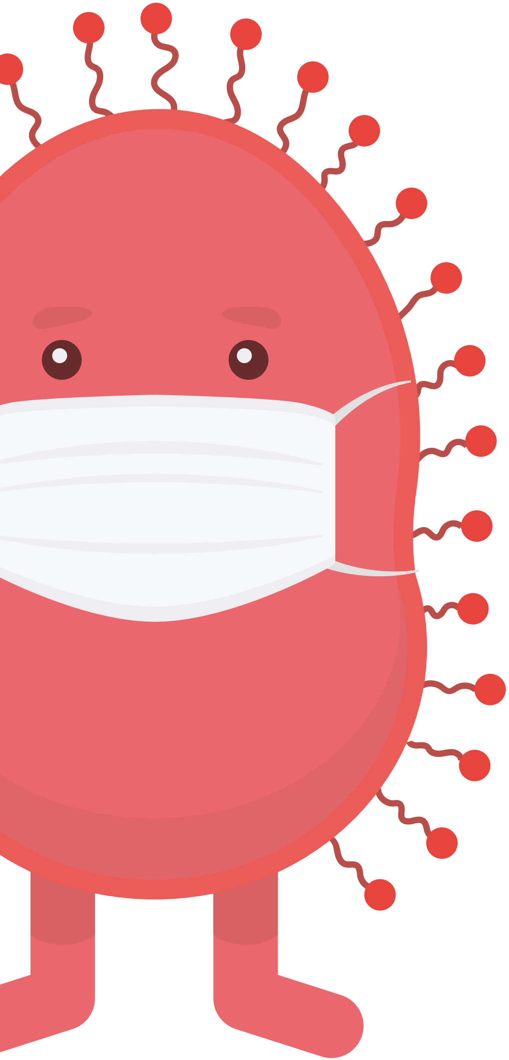

Vous en souvenez-vous ?

Lors de votre visite médicale d'engagement à l'Unité de médecine du personnel et d'entreprise, nous avions discuté des avantages de la vaccination contre la grippe.

La campagne de vaccination est désormais ouverte, alors… faites le bon choix !

SAM | CHUV 2016 | 19820

canton de vaud LIBERTÉ ET PATRIE
