# Supplementary material for: Promotion of the influenza vaccination to hospital staff during pre-employment health check: a prospective, randomised, controlled trial
Source: J Occup Med Toxicol. 2020 Nov 18;15:34. doi: 10.1186/s12995-020-00285-w (PMC7672907; doi:10.1186/s12995-020-00285-w)
Supplement: Supplementary file 3 — Additional file 3. Exploratory comparisons in other subgroups. [file 12995_2020_285_MOESM3_ESM.pdf]

Exploratory comparisons in other subgroups

Subgroup	Characteristic, n	Not vaccinated (%)	Vaccinated (%)	p
Patient-care employees				
	Intervention: No, 72	39 (54)	33 (46)	
	Intervention: Yes, 86	44 (51)	42 (49)	0.750
	Mean age (years)	32	31	0.751
	Sex: males, 36	22 (61)	14 (39)	
	Sex: females, 122	61 (50)	61 (50)	0.260
	Single: No, 126	69 (55)	57 (45)	
	Single: Yes, 32	14 (44)	18 (56)	0.323
	Swiss citizen: No, 74	35 (47)	39 (53)	
	Swiss citizen: Yes, 84	48 (57)	36 (43)	0.264
	Allergy: No, 111	61 (55)	50 (45)	
	Allergy: Yes, 47	22 (47)	25 (53)	0.386
	Asthma: No, 150	81 (54)	69 (46)	
	Asthma: Yes, 8	2 (25)	6(75)	0.151
	New employee: No, 75	42 (56)	33 (44)	
	New employee: Yes, 83	41 (49)	42 (51)	0.429
	Undergoing training: No, 151	79 (52)	72 (48)	
	Undergoing training: Yes, 7	4 (57)	3 (43)	1
Female sex				

	Intervention: No, 118	61 (52)	57 (48)	
	Intervention: Yes, 139	73 (53)	66 (47)	0.901
	Mean age (years)	32	32	0.724
	Single : No, 199	106 (53)	93 (47)	
	Single: Yes, 58	28 (48)	30 (52)	0.552
	Swiss citizen: No, 133	70 (52)	63 (47)	
	Swiss citizen: Yes, 124	64 (52)	60 (48)	0.901
	Allergy : No, 186	102 (55)	84 (45)	
	Allergy: Yes, 71	32 (45)	39 (55)	0.166
	Asthma: No, 247	132 (53)	115 (47)	
	Asthma: Yes, 10	2 (20)	8 (80)	0.052
	New employee: No, 112	63 (56)	49 (44)	
	New employee: Yes, 145	71 (52)	74 (51)	0.259
	Undergoing training: No, 239	121 (51)	118 (49)	
	Undergoing training: Yes, 18	13 (72)	5 (28)	0.090
	Physician: No, 193	108 (56)	85 (44)	
	Physician: Yes, 64	26 (41)	38 (59)	0.043
	Patient-care employees: No, 135	73 (54)	62 (46)	
	Patient-care employees: Yes, 122	61 (50)	61 (50)	0.534
Allergic				
	Intervention: No, 49	25 (51)	24 (49)	

	Intervention: Yes, 52	22 (42)	30 (57)	0.428
	Mean age (years)	32	33	0.682
	Sex: males, 30	15 (50)	15 (50)	
	Sex: females, 71	32 (45)	39 (55)	0.669
	Single: No, 77	39 (51)	38 (49)	
	Single: Yes, 24	8 (33)	16 (67)	0.164
	Swiss citizen: No, 59	27 (46)	32 (54)	
	Swiss citizen: Yes, 42	20 (48)	22 (52)	1
	Asthma: No, 83	43 (52)	40 (48)	
	Asthma: Yes, 18	4 (22)	14 (78)	0.035
	New employee: No, 52	26 (50)	26 (50)	
	New employee: Yes, 49	21 (43)	28 (57)	0.551
	Undergoing training: No, 96	44 (46)	52 (54)	
	Undergoing training: Yes, 5	3 (60)	2 (40)	0.661
	Physician: No, 70	40 (57)	30 (43)	
	Physician: Yes, 31	7 (23)	24 (77)	0.002
	Patient-care employees: No, 54	25 (46)	29 (54)	
	Patient-care employees: Yes, 47	22 (47)	25 (53)	1
Asthma				
	Intervention: No, 11	5 (45)	6 (55)	
	Intervention: Yes, 9	1 (11)	8 (89)	0.157

	Mean age (years)	32	31	0.532
	Sex: males, 10	4 (40)	6 (60)	
	Sex: females, 10	2 (20)	8 (80)	0.628
	Single: No, 14	5 (36)	9 (64)	
	Single: Yes, 6	1 (17)	5 (83)	0.613
	Swiss citizen: No, 17	6 (35)	11 (65)	
	Swiss citizen: Yes, 3	0 (0)	3 (100)	0.521
	Allergy: No, 2	2 (100)	0 (0)	
	Allergy: Yes, 18	4 (22)	14 (78)	0.079
	New employee: No, 11	4 (36)	7 (64)	
	New employee: Yes, 9	2 (22)	7 (78)	0.642
	Undergoing training: No, 20	6 (30)	14 (70)	
	Undergoing training: Yes, 0	0 (0)	0 (0)	
	Physician: No, 12	5 (42)	7 (58)	
	Physician: Yes, 8	1 (12)	7 (88)	0.325
	Patient-care employees: No, 12	4 (33)	8 (67)	
	Patient-care employees: Yes, 8	2 (25)	6 (75)	1
New employee				
	Intervention: No, 81	38 (47)	43 (53)	
	Intervention: Yes, 106	51 (48)	55 (52)	0.884

	Mean age (years)	31	31	0.193
	Sex: males, 42	18 (43)	24 (57)	
	Sex: females, 145	71 (49)	74 (51)	0.599
	Single: No, 146	72 (49)	74 (51)	
	Single: Yes, 41	17 (41)	24 (59)	0.384
	Swiss citizen: No, 78	44 (56)	34 (44)	
	Swiss citizen: Yes, 109	45 (41)	64 (59)	0.053
	Allergy: No, 138	68 (49)	70 (51)	
	Allergy: Yes, 49	21 (43)	28 (57)	0.507
	Asthma: No, 178	87 (49)	91 (51)	
	Asthma: Yes, 9	2 (22)	7 (78)	0.174
	Undergoing training: No, 171	76 (44)	95 (56)	
	Undergoing training: Yes, 16	13 (81)	3 (19)	0.007
	Physician: No, 140	78 (56)	62 (44)	
	Physician: Yes, 47	11 (23)	36 (77)	0.000
	Patient-care employees: No, 104	48 (46)	56 (54)	
	Patient-care employees: Yes, 83	41 (49)	42 (51)	0.768
Undergoing training				
	Intervention: No, 6	5 (83)	1 (17)	
	Intervention: Yes, 15	11 (73)	4 (27)	1
	Mean age (years)	25	21	0.0128

	Sex: males, 3	3 (100)	0 (0)	
	Sex: females, 18	13 (72)	5 (28)	0.549
	Single: No, 21	16 (76)	5 (24)	
	Single: Yes, 0	0 (0)	0 (0)	
	Swiss citizen: No, 15	12 (80)	3 (20)	
	Swiss citizen: Yes, 6	4 (67)	2 (33)	0.598
	Allergy: No, 16	13 (81)	3 (19)	
	Allergy: Yes, 5	3 (60)	2 (40)	0.553
	Asthma: No, 21	16 (76)	5 (24)	
	Asthma: Yes, 0	0 (0)	0(0)	
	New employee: No, 5	3 (60)	2 (40)	
	New employee: Yes, 16	13 (81)	3 (19)	0.553
	Physician: No, 20	15 (75)	5 (25)	
	Physician: Yes, 1	1 (100)	0 (0)	1
	Patient-care employees: No, 14	12 (86)	2 (14)	
	Patient-care employees: Yes, 7	4 (57)	3 (43)	0.280
